# Health Impact Assessment: A Missed Opportunity for MCH Professionals in Their Quest to Address the Social Determinants of Health

**DOI:** 10.1007/s10995-021-03350-w

**Published:** 2022-01-24

**Authors:** James E. Dills, Taylor M. Lawson, Jane Branscomb, Amy Mullenix, Kristen Hassmiller Lich

**Affiliations:** 1 Georgia Health Policy Center, Andrew Young School of Policy Studies, Georgia State University, 55 Park Place NE, 8th Floor, Atlanta, GA 30303 USA; 2 Rollins School of Public Health, Emory University, Grace Crum Rollins Building, 1518 Clifton Road, Atlanta, GA 30322 USA; 3 Gillings School of Global Public Health, University of North Carolina - Chapel Hill, 412 Rosenau Hall CB #7445, Chapel Hill, NC 27599 USA; 4 Gillings School of Global Public Health, University of North Carolina - Chapel Hill, 1105E McGavran-Greenberg Hall CB #7411, Chapel Hill, NC 27599 USA

**Keywords:** Social determinants of health, Equity, Policy, Maternal and child health workforce, Health impact assessment, Health in all policies, Systems integration

## Abstract

**Introduction:**

Public health professionals, especially ones concerned with maternal and child health (MCH), need to engage in cross-sector collaborations to address social determinants of health. Health Impact Assessment (HIA) systematically brings public health perspectives into non-health decision-making contexts that influence social determinants. Alignment of MCH and HIA practice has not previously been documented.

**Methods:**

An exploratory review of HIAs conducted in the United States considered several dimensions of MCH-HIA alignment and produced data to test the hypothesis that HIAs involving MCH stakeholders are more likely to address MCH populations and relevant measures. The review examined three key variables for each HIA: inclusion of MCH-focused stakeholders, level of focus on MCH populations, and presence of MCH-relevant content.

**Results:**

Of the 424 HIAs included in the database of US HIAs, 350 were included in this review. Twenty-four percent (84) included MCH-focused stakeholders, and 42% (148) focused on MCH populations. Ninety percent (317) included metrics or content relevant to at least one Title V National Performance Measure (NPM). HIAs that clearly included MCH stakeholders had seven times the odds of including both a focus on MCH populations and at least one NPM-relevant topic compared to HIAs that did not clearly include MCH stakeholders (OR 6.98; 95% CI 3.99, 12.20).

**Discussion:**

Despite low engagement of MCH stakeholders in HIAs, many still consider MCH populations and measures. Intentional engagement of MCH workforce in HIAs could ensure greater alignment with existing MCH priorities (such as addressing the social determinants of health and equity) in a given jurisdiction.

## Significance Statement

*What is already known on this subject?* Cross-sector collaboration has become increasingly important for public health practice. HIA is an effective tool for building collaborative relationships across sectors. The MCH workforce is in need of actionable frameworks for cross-sector collaboration to impact social determinants of health and equity.

*What this study adds?* HIA practice in the US has not seen extensive involvement of the MCH workforce, but it has regularly incorporated MCH-relevant content. By filling this gap and becoming more involved in HIA, MCH professionals have an opportunity to build cross-sector capacity and ensure relevance of MCH content.

## Introduction

Researchers, practitioners, and policy-makers need to address social determinants of health to meaningfully reduce population-level disparities and improve maternal and child health (MCH). Social determinants of health are conditions in the environments where people are born, live, learn, work, play, worship, and age that affect a wide range of health, functioning, and quality-of-life outcomes and risks (U.S. Department of Health and Human Services Office of Disease Prevention and Health Promotion, 2018). Addressing disparities requires a commitment to health equity, which means striving for the highest possible standard of health for all people and giving special attention to needs of individuals at greatest risk based on social conditions (Braveman, 2014). The Maternal and Child Health Bureau of the Health Resources and Services Administration has emphasized a need for integrating perspectives of social determinants and equity into MCH practice (Fine et al., 2010). Meeting this need requires collaboration between public health and other sectors like education, housing, and transportation (DeSalvo et al., 2016; Koh et al., 2011; Mattessich & Rausch, 2014).

Research on training needs of the public health workforce, and the MCH workforce specifically, emphasizes policy engagement and collaboration with sectors outside traditional public health silos as areas for improvement (Bogaert et al., 2019; DeSalvo et al., 2017; Raskind et al., 2019; Sellers et al., 2015). As a result, the National MCH Workforce Development Center advances strategies to help Title V leaders and MCH practitioners build collaborative policy engagement capacity, using systems integration perspectives that emphasizes social determinants of health and equity (Clarke & Cilenti, 2018; Margolis et al., 2017). One of these strategies is implementing a Health in All Policies (HiAP) approach.

HiAP considers how to systematically integrate public health perspectives on social determinants and equity into decision-making across non-health sectors (Ståhl et al., 2006; Wernham & Teutsch, 2015). One of the more common HiAP approaches is health impact assessment (HIA) (Gase et al., 2013; Rudolph et al., 2013). The National Research Council defines HIA as:

A systematic process that uses an array of data sources and analytic methods and considers input from stakeholders to determine the potential effects of a proposed policy, plan, program, or project on the health of a population and the distribution of those effects within the population. HIA provides recommendations on monitoring and managing those effects (2011).

While variations exist in practice, conducting HIA typically involves the iterative six-step process illustrated in Table 1 and components of the eight Minimum Elements listed in Table 2 (Bhatia et al., 2014; Dannenberg, 2016a; National Research Council, 2011).

**Table 1:** Six steps of health impact assessment and associated outputs (National Research Council, 2011)

Step	Outputs
Screening	Describes proposed policy, program, plan, or project, including timeline for decision and political and policy context Presents preliminary opinion on importance of proposal for health and the opportunities for HIA to inform the decision, and states why the proposal was selected for screening Outlines expected resource requirements to conduct HIA Provides recommendation on whether HIA is warranted
Scoping	Summarizes pathways and health effects to be addressed, and provides rationale for those included and excluded Identifies affected populations and vulnerable groups Describes research questions, data sources, the analytic plan, data gaps, and how gaps will be addressed Identifies alternatives to the proposed action to be assessed Summarizes stakeholder engagement, issues raised by stakeholders, and responses to those issues
Assessment	Describes the baseline health status of affected populations Analyzes and characterizes beneficial and adverse health effects of the proposal and each alternative Describes data sources and analytic methods used Documents stakeholder engagement and integrates input into analyses Identifies clearly the limitations and uncertainties of the analysis
Recommendations	Identifies alternatives to proposal or actions that could be taken to avoid, minimize, or mitigate adverse effects and to optimize beneficial ones Proposes a health-management plan to identify stakeholders who could implement recommendations, indicators for monitoring, and systems for verification
Reporting	Provides clear documentation of the proposal analyzed, the population affected, stakeholder engagement, data sources and analytic methods used, findings, and recommendations Communicates findings and recommendations to decision makers, the public, and other stakeholders in a form that can be integrated with other decision-making factors (technical, social, political, and economic)
Monitoring & Evaluation	Tracks changes in health indicators or implementation of HIA recommendations Evaluates (a) whether the HIA was conducted according to its plan and applicable standards (process evaluation), (b) whether the HIA influenced the decision-making process (impact evaluation), and (c) when practicable, whether implementation of the proposal changed health indicators (outcome evaluation)

**Table 2:** Eight minimum elements of health impact assessments (Bhatia et al., 2014)

1. HIA is conducted to assess the potential health consequences of a proposed program, policy, project, or plan under consideration by decision-makers, and is conducted in advance of the decision in question
2. HIA involves and engages stakeholders affected by the proposal, particularly vulnerable populations
3. HIA systematically considers the full range of potential impacts of the proposal on health determinants, health status, and health equity
4. HIA provides a profile of existing conditions for the populations affected by the proposal, including their health outcomes, health determinants, and vulnerable sub-groups within the population, relevant to the health issues examined in the HIA
5. HIA characterizes the proposal’s impacts on health, health determinants, and health equity, while documenting data sources and analytic methods, quality of evidence used, methodological assumptions, and limitations
6. HIA provides recommendations, as needed, on feasible and effective actions to promote the positive health impacts and mitigate the negative health impacts of the decision, identifying, where appropriate, alternatives or modifications to the proposal
7. HIA produces a publicly accessible report that includes, at minimum, documentation of the HIA’s purpose, findings, and recommendations, and either documentation of the processes and methods involved, or reference to an external source of documentation for these processes and methods. The report should be shared with decision-makers and other stakeholders
8. HIA proposes indicators, actions, and responsible parties, where indicated, for a plan to monitor the implementation of recommendations, as well as health effects and outcomes of the proposal

HIA is a practical tool for synthesizing evidence and stakeholder input to foster collaboration and inform decisions made outside the health sector (Cole & Fielding, 2007). Because individual HIAs are primarily oriented toward practice, they are under-documented in peer-reviewed research literature. HIA researchers launched a dedicated journal in 2016 to address this issue and to provide more opportunities for HIA practitioners to publish their results (Stone, 2016). Over the past decade, published research on HIA in the United States has mostly considered effectiveness of the process, while individual case studies remain elusive in traditional research databases (Cole et al., 2019; Dannenberg, 2016b; Sohn et al., 2018). For example, searching the key words “health impact assessment” in PubMed returns a large number of results, but most are not specific to the prospective process defined above.

A PubMed search for “health impact assessment” conducted by the authors in January 2020 returned 1,264 results. When sorted by relevance, the first 100 results included 19 published accounts of specific HIAs, of which five were conducted in the United States. In the MCH context, even fewer research publications regarding HIA are available: as of January 2020, the Maternal and Child Health Journal has published no articles describing “Health Impact Assessment” in their title or abstracts. To the authors’ knowledge, the current study is the first of its kind to document potential for alignment between MCH and HIA practice in the United States and to highlight the breadth of opportunities for MCH practitioners to engage in such HiAP initiatives as a strategy to address the social determinants of health.

## Methods

### Research Overview and Key Questions

**Fig. 1:**
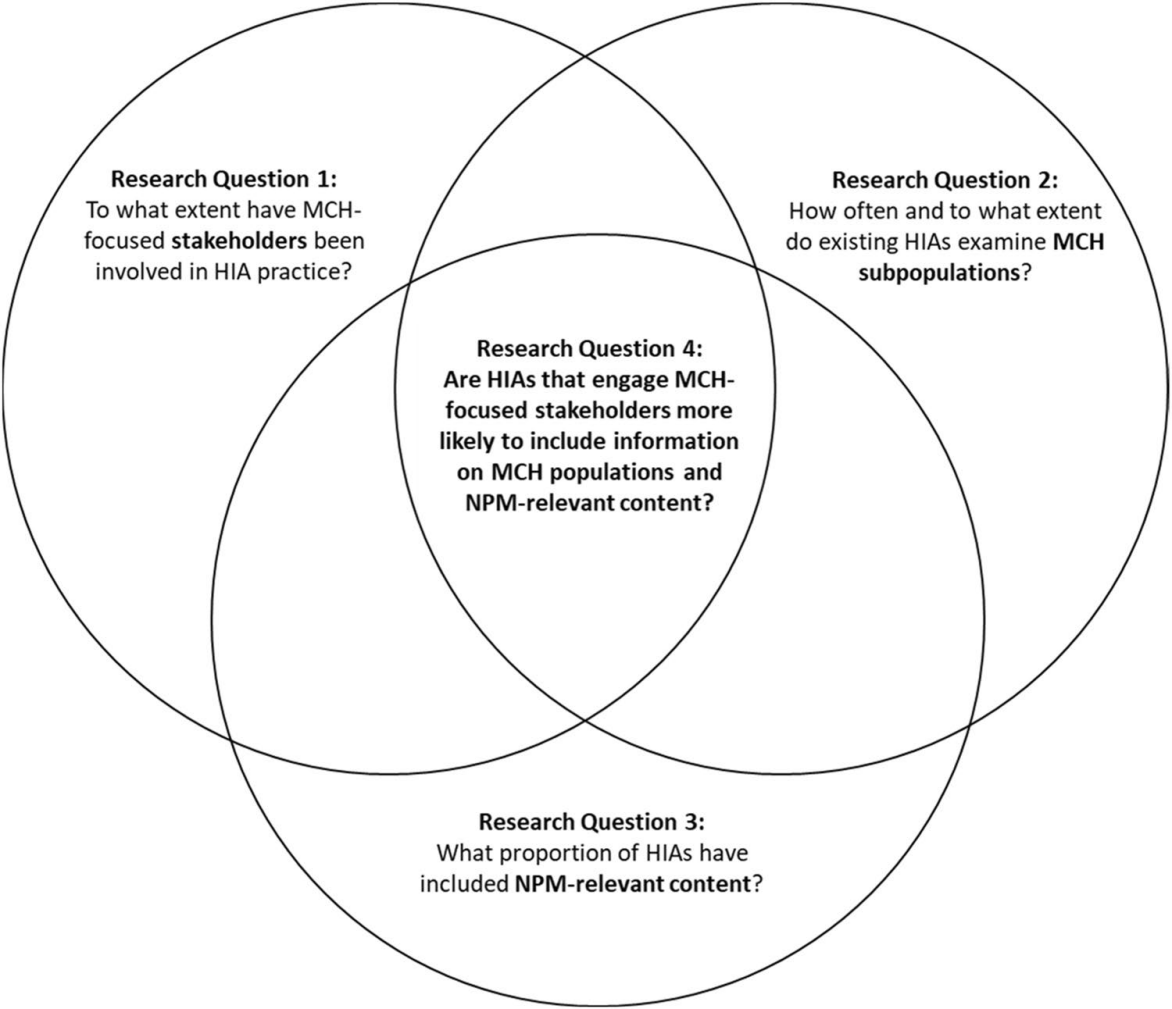
Interrelatedness of four research questions guiding the review of HIAs

This study leveraged a repository of HIAs conducted in the United States to answer these questions: (1) to what extent have MCH-focused stakeholders been involved in HIA practice? (2) How often and to what extent do HIAs examine MCH subpopulations? (3) What proportion of HIAs include Title V National Performance Measures (NPMs) or closely related metrics in their analyses? (4) Are HIAs that engage MCH-focused stakeholders more likely to include information on MCH populations and NPM-relevant content? Fig. 1 illustrates the interrelatedness of the four research questions. Collected data also characterize the breadth of non-health policies relevant to MCH outcomes and a range of opportunities for MCH stakeholders to inform them. This study does not include any clinical or patient data.

### Data Source and Inclusion Criteria

The Health Impact Project, a collaboration of the Robert Wood Johnson Foundation and The Pew Charitable Trusts, hosts a web-based repository of information about HIAs conducted in the United States (Health Impact Project, 2017). HIA researchers commonly use this repository as a data source (Cole et al., 2019; Cowling et al., 2017; Dannenberg et al., 2019; Gase et al., 2017). Although uploading HIA information to the site is voluntary, this repository has been viewed as a largely complete picture of US practice since its inception in 2010.

As of September 2017, the repository listed 424 HIAs. The entry for each HIA includes hyperlinks and contextual data like project title, lead organization(s), decision-making level (local, regional, state, or federal), and target sector (e.g. housing, education, transportation, etc.). HIAs were excluded from the current analysis when project documentation was not available, either because the HIA was in progress or because available hyperlinks were no longer active and reviewers were unable to find publicly available documentation via web search based on HIA title and/or lead organization.

### Key Variables

Researchers coded three primary variables for each HIA reviewed. First, they determined the *involvement of MCH-focused stakeholders*. MCH-focused stakeholders included organizations or individuals that focus on infants, children, youth, adolescents, mothers, pregnant individuals, and/or families. State or Local Health Departments were only included as MCH-focused stakeholders if available documentation clearly noted representation from an MCH-serving division, branch, or team (e.g. the Title V Office, Division of Child Health, etc.). Reviewers did not assume inclusion of these perspectives unless stated. This variable was coded ‘yes’ if documentation revealed an explicit role for an MCH-focused partner in the conduct of the HIA, ranging from roles on the HIA project team or advisory committee to participation in document review or data collection as part of the process. If available documentation mentioned MCH-focused stakeholders but was unclear about the extent of involvement, reviewers coded this variable as ‘partial.’ The ‘partial’ designation was also used when the reviewer was unsure about the extent to which a given stakeholder explicitly focused on MCH, for example local educational institutions were regularly placed in this category.

The second variable considers *attention given to MCH populations* within the HIA. MCH populations include infants, children, youth, adolescents, mothers, pregnant individuals, and/or families. Reviewers coded this variable as ‘yes’ if documentation noted explicit focus on one or more of these MCH populations as part of the HIA scope. This meant that the HIA not only identified an MCH population as a subpopulation of concern, but that the assessment and/or recommendations reflect elevated attention on that group. Reviewers assigned a ‘partial’ designation if the HIA mentioned MCH populations but did not clearly incorporate them as part of the assessment. This included instances where a report includes statistics on an MCH population but not as a critical element of the analysis (e.g. the childhood obesity rate was mentioned as part of existing conditions but not in the assessment of potential impacts).

To define the third key variable, *inclusion of MCH content*, reviewers determined whether each HIA included topics related to one or more of the Title V NPMs. These 15 measures were selected as the basis for defining "MCH content" in this review because national leaders have identified them as foundational to the work of public health MCH professionals, as measurable, as directly modifiable by states’ programmatic activities, and as meaningful when changed (Kogan et al., 2015). Specific guidance and search terms were developed for each NPM and are listed in Table 3, along with examples of reviewer notes from specific HIAs. Reviewers only used the ‘yes’ designation when the exact NPM was noted in the HIA. They used the ‘partial’ designation when an NPM topic was touched upon in the HIA, but without including the exact NPM. A goal of this review is to inspire MCH professionals toward more upstream cross-sector engagement and collaboration by documenting the breadth of opportunities where their perspectives could have been valuable in existing HIA practice. Because this retrospective accounting of opportunities was generated from content positioned primarily within non-MCH perspectives, the authors were intentionally inclusive in identifying aspects of a given HIA that could be considered MCH-relevant content, and thus, some instances may stretch the bounds of what is considered closely related to specific NPMs.

**Table 3:** Specific guidance for HIA reviewers used to characterize NPM inclusion

Title V National Performance Measure (NPM)	HIA Review search terms and coding guidance
NPM 1: Well-Woman Visit (Percent of women with a past year preventive medical visit)	Search Terms: women, woman, preventive Guidance to Reviewers: This NPM is concerned with preventive care for women. HIAs may include other measures of preventive care use by women, and instances should be included and explained as part of the review Example from Review Data: "Among women, close to half (47.7%) indicated that they had visited an obstetrician or gynecologist in the past year” (p. 62).—*A Health Impact Assessment of the Healthy Families Act of 2009* (Federal)
NPM 2: Low-Risk Cesarean Delivery (Percent of cesarean deliveries among low-risk first births)	Search Terms: birth, Cesarean Guidance to Reviewers: This NPM considers Low-risk Cesarean delivery and did not come into play during the pilot reviews. Any instance of an HIA mentioning Cesarean births should be included in order to prompt further discussion Example from Review Data: Maternity leave (paid) for women who have a cesarean birth (Table 2 on page 17). Also see "Health care providers, however, strongly believe that at a very minimum 6 weeks is required for a mother to recover from a normal childbirth and up to 3 months is required after a complicated or caesarian delivery" (p. 30).—*Pathways to a Healthy Decatur:* *A Rapid Health Impact Assessment of the City of Decatur Community Transportation Plan* (Georgia)—2007
NPM 3: Risk-Appropriate Perinatal Care (Percent of very low birth weight (VLBW) infants born in a hospital with a Level III + Neonatal Intensive Care Unit (NICU))	Search Terms: perinatal, birth, weight, birthweight Guidance to Reviewers: A number of HIAs are likely to note low birth weight as an outcome associated with (most commonly) air pollution. While this type of information is not exactly the same as that describing the care received by very low birth weight infants, as reflected in the NPM (Risk-Appropriate Perinatal Care), noting any consideration of birthweight in this column is still important for the purpose of this review Example from Review Data: Low birth weight and preterm births are discussed multiple times throughout the HIA, particularly in connection to food insecurity and unhealthy eating for pregnant individuals. Tables and analysis provide information on low birth weight and preterm birth, specifically.—*A Health Impact Assessment of a Food Tax in New Mexico: An analysis of the taxation of grocery purchases and its impacts on the health of the state’s children, families, and communities*—2015)
NPM 4: Breastfeeding (A) Percent of infants who are ever breastfed and (B) Percent of infants breastfed exclusively through 6 months)	Search Terms: breast Guidance to Reviewers: Breastfeeding did not come up in any of the pilot HIAs and is likely to be rare in the larger sample, so be inclusive of any mention of breastfeeding for this review Example from Review Data: "Barriers to breastfeeding among WIC participants have included sore nipples and pain, perceptions of inadequate milk supply, and social support networks' attitudes about breastfeeding. It is possible that these barriers would be heightened for homeless women because of inadequate access to lactation resources, concerns about inadequate milk production, or because of returning to the workforce" (p. 105).—*planokc Comprehensive Plan* *Health Impact Assessment* (Oklahoma City)—2014
NPM 5: Safe Sleep (Percent of infants placed to sleep on their backs)	Search Terms: sleep, infant Guidance to Reviewers: Some HIAs in the pilot noted a connection between health, sleep, and noise exposure. The larger review is therefore expected to encounter this issue again. In cases where sleep and/or sleep disturbance are considered, only include under this column if documentation notes a specific tie to infants. The NPM is concerned with a specific safe sleep practice (percent of infants placed to sleep on their backs), and while we anticipate few (if any) HIAs will directly consider this indicator, it will be useful to consider ones that touch on infant sleep at all as getting ‘close’ to this measure Example from Review Data: "Aircraft noise in the late evening has also been shown to disturb sleep among adults and infants" (p. 26).—*Health Impact Assessment (HIA) of Cargo Atlanta: a Citywide Freight Study*—2016
NPM 6: Developmental Screening (Percent of children, ages 10 through 71 months, receiving a developmental screening using a parent-completed screening tool)	Search Terms: Developmental, Screening. Note: ‘Screening’ is likely to generate false positives because it describes a step in the HIA process Guidance to Reviewers: This NPM focuses on developmental screening of youth up through age 5 (71 months) and appears unlikely to be directly addressed in existing HIAs; however, there may be instances where the physical and/or mental development of young children get discussed in an HIA. In these instances, be inclusive so we can return to them for further discussion Example from Review Data: "PCBs and mercury. Human consumption of these fish can lead to chronic health problems, and children are at the greatest risk for developmental effects from such exposure." (p. 52). Development is also discussed in connection with parks/green spaces, particularly for children with ADHD.—*SR 520 Health Impact Assessment: A bridge to a healthier community* (Seattle)—2008
NPM 7: Injury Hospitalization (Rate of hospitalization for non-fatal injury per 100,000 children ages 0 through 9 and adolescents 10 through 19)	Search Terms: injury, injuries, safe Guidance to Reviewers: Injuries will be one of the more common topics included in HIAs, especially ones focused on transportation-related decisions. Include any mention of statistics related to injury (mortality and morbidity; intentional and unintentional) here and note where youth injuries are called out specifically. To be inclusive, also consider when perceptions of injury risk are being discussed. Likely to get very ‘close’ on this NPM, but only count as ‘yes’ over ‘partial’ if all the exact components of the NPM are noted: Rate of hospitalization for non-fatal injury per 100,000 children ages 0 through 9 and adolescents 10 through 19 Example from Review Data: "In addition, among accidental injuries that result in death for Baltimore children ages 1–17, 39% are motor vehicle-related. Nearly two-thirds of these deaths involved a child who was walking or biking" (p. 16).—*The Red Line Transit Project Health Impact Assessment* (Baltimore)—2008
NPM 8: Physical Activity (Percent of children ages 6 through 11 and adolescents 12 through 17 who are physically active at least 60 min per day)	Search Terms: physical activity, children Guidance to Reviewers: Physical activity (PA) will also be among the most common topics addressed in the HIAs, allowing for us to be a little more conservative on inclusion. When PA statistics are given that would cover children or youth (anyone under age 19), include it as at least a ‘partial’. There may be instances where all the data presented are for adult PA, but the discussion includes youth promotion. Capture these instances as a partial with explanation here and/or in the columns considering inclusion of MCH populations. If the HIA is only focused on adult PA and has no mention of the non-adult population, mark it as a ‘no’. As with the injury NPM, HIAs are expected to get very ‘close’ on this NPM, but only denote it as a ‘yes’ over a ‘partial’ if all the exact components of the NPM are noted: percent of children ages 6 through 11 and adolescents 12 through 17 who are physically active at least 60 min per day Example from Review Data: "Children who began and maintained physical activity levels into young adulthood had better mental health outcomes than children who were inactive or those who did not maintain physical activity levels" (p. 15).—*Winona County Active Living Plan HIA* (Minnesota)—2015
NPM 9: Bullying (Percent of adolescents, ages 12 through 17, who are bullied or who bully others)	Search Terms: bully Guidance to Reviewers: In the pilot, at least one HIA came up for discussion under this NPM. While it did not directly note bullying or measures thereof, the assessment did spend some time discussing possible impacts on student behavior/disruption in school, which could include bullying. To be inclusive, use your judgement as to whether or not any mentions of youth behavior in school or other social settings might include bullying Example from Review Data: "Studies that have examined how social capital is reflected in children's health and educational outcomes suggest the effect is positive on behavior problems" (p. 43). Also—"Another study notes that among victims of child maltreatment, psychological problems are prevalent and often manifest in aggressive behaviors towards both adults and peers," (p. 44).—*Transitional Jobs Programs: A Health Impact Assessment* (Wisconsin)—2013
NPM 10: Adolescent Well-Visit (Percent of adolescents, ages 12 through 17, with a preventive medical visit in the past year)	Search Terms: well-visit, adolescent Guidance to Reviewers: Similar to NPM 1, this NPM is focused on preventive care—in this case for youth. While no instances occurred in the pilot, there may be times when data on aspects of youth prevention are included. Use your judgement, but err on the side of inclusivity since presence of this topic is likely to be a rare instance within the HIAs Example from Review Data: Access to healthcare for children and adolescents—"More broadly, in a comparison study of schools with and without [school-based health centers], [Denver Health’s School-Based Health Centers] located within school were more effective at reducing barriers to care and increasing access to and use of health care services for children and adolescents" (p. 21).—*Addressing Mental Health and Physical Activity in K-12 Children in Colorado Springs: A Health Impact Assessment*—2016
NPM 11: Medical Home (Percent of children with and without special health care needs having a medical home)	Search Terms: medical home, special needs Guidance to Reviewers: This NPM is unlikely to be directly addressed in many HIAs, but topics like youth insurance coverage and health care utilization might arise. Use your judgement and err on the side of inclusiveness, but only when children/youth are explicitly considered. Also relevant here are youth with special needs. Include the mention of children and youth with special needs under the MCH Populations columns unless the HIA explicitly discusses access to health care for that group, in which case it would fit under this NPM discussion as well Example from Review Data: "In North Carolina, children with disabilities are classified as children with special health care needs (CSHCN), defined as ‘children who need prescription medications or have an elevated need for medical, mental health, or education services due to a medical, behavioral, or other health condition that has lasted or is expected to last for at least 12 months.’ According to results of the 2011 NC Child Health Assessment and Monitoring Program (CHAMP), 18% of children ages 0–17 are considered CSHCN. Using this percentage and 2010 U.S. Census data (2,500 children in Davidson), approximately 450 children within Davidson would be considered CSHCN" (p. 8).—*Universal Design in Single-Family Housing: A Health Impact Assessment (HIA) in Davidson, NC*—2013
NPM 12: Transition (Percent of adolescents with and without special health care needs who received services necessary to make transitions to adult health care)	Search Terms: transition, adolescent, special needs Guidance to Reviewers: (Similar to NPM 11) This NPM is unlikely to be directly addressed in many HIAs, but topics like youth insurance coverage and health care utilization might arise. Use your judgement and err on the side of inclusiveness, but only when older youth are explicitly considered. Also relevant here are youth with special needs. Include the mention of children and youth with special needs under the MCH Populations columns unless the HIA explicitly discusses access to health care for that group, in which case it would fit under this NPM discussion if age-specific aspects are considered Example from Review Data: "Assure the availability of a medical home for all to increase age-specific health screening and preventative care" (p. 23).—*Buncombe County Greenways & Trails Master Plan Health Impact Assessment* (North Carolina)—2013
NPM 13: Preventive Dental Visit (A) Percent of women who had a dental visit during pregnancy and (B) Percent of children, ages 1 through 17 who had a preventive dental visit in the past year)	Search Terms: dental, dentist, oral, oral health Guidance to Reviewers: Few HIAs are likely to include oral health, much less for women and children specifically. To be inclusive, note any references to oral health found during your search, as ‘partial’ with explanation to inform future discussion Example from Review Data: "According to a survey performed by the National Energy Assistance Directors Association (NEADA) in 2005, a significant proportion of LIHEAP participants in the Northeast reported making precisely these kinds of budget trade-offs due to high energy costs:... 28% went without medical or dental care;" (pp. 2–3). Also see "Housing instability and homelessness pose well-documented threats to child physical health—have 10 times more dental caries than housed children" (p. 8).—*Unhealthy Consequences: Energy Costs and Child Health|A Child Health Impact Assessment of Energy Costs and the Low Income Home Energy Assistance Program* (Massachusetts)—2007
NPM 14: Smoking (A) Percent of women who smoke during pregnancy and (B) Percent of children who live in households where someone smokes)	Search Terms: smoke, smoking, tobacco Guidance to Reviewers: This topic did not come up in the pilot reviews. Include instances that consider smoking and second-hand smoke as ‘partial’ and explain Example from Review Data: "Parks and recreational facilities should be a smoke-free environment to reduce exposure to secondhand smoke − especially for youth who respire more frequently than adults while being physically active" (p. 10).—"One in five children is exposed to secondhand smoke in cars. Switching from car to bus, where smoking is not allowed, could help decrease children’s exposure to secondhand smoke" (p. 23).—*Potential Health Effects of Proposed Public Transit Concepts in Wichita, Kansas*—2013
NPM 15: Adequate Insurance (Percent of children ages 0 through 17 who are adequately insured)	Search Terms: insurance, insured Guidance to Reviewers: If the HIA talks about how a decision could influence the rate of individuals or families that have insurance, be inclusive. Highlight when/if the discussion is specific to youth Example from Review Data: "Most students receive medical insurance via Medicaid (93%), while 2% have private insurance and 5% of the students have no form of insurance at all." (p. 8).—*Health Impact Assessment of the Demolition of a Lead Painted Bridge Adjacent to a Residential Area* (Cincinnati)—2013

### Review and Extraction Process

Starting with information available in Health Impact Project’s repository, researchers followed a protocol created and pilot-tested specifically for this review. First, they used project hyperlinks to obtain publicly available HIA documentation. This documentation was then reviewed to determine designations for the three key variables described above. In addition to a scan of available documentation for each HIA, variable-specific search terms were employed to more efficiently identify relevant content. Reviewers extracted this relevant content and summarized how it supported designations of ‘yes’, ‘partial’, or ‘no’ for each variable. A pilot-test of the protocol on 20 randomly selected HIAs included independent review of each by three researchers, who agreed 85% of the time across all variable designations. No HIA in the pilot explicitly included any of the 15 NPMs, which led researchers to adjust the final review protocol for this variable to be more inclusive of related content within the ‘partial’ designation. Two co-authors then collaboratively reviewed the remaining HIAs and adjudicated disagreements through discussion as needed. When reviewers could not make a clear determination, the research team made a final coding determination based on collective interpretation of the available content in the context of the study objectives. Reviewers applied the protocol to available materials and coded responses into an Excel spreadsheet.

### Analysis

Coded data were transferred to SPSS (IBM Corp., 2017), and frequency tables were produced for each research question. Upon initial review of the data, yes/partial/no responses were recoded to facilitate statistical analysis and hypothesis testing. For the MCH stakeholder and MCH population variables, ‘no’ and ‘partial’ responses were combined to allow dichotomous comparison to the ‘yes’ responses. Because so few HIAs were coded as ‘yes’ for including NPMs, responses for this variable were dichotomized by combining ‘yes’ and ‘partial’ designations for comparison with those HIAs designated as ‘no.’ Researchers also created a variable that indicated the total number of NPMs each HIA addressed, ranging from 0 to 15.

To test the hypothesis that HIAs with MCH stakeholder involvement are more likely to include a focus on MCH populations and NPM-related topics, a single dichotomous dependent variable was created that combined inclusion of MCH populations and NPM-relevant topics. These binary data were used to conduct Chi-Square and Simple Odds Ratios to test the hypothesis. Statistical significance was determined at alpha = 0.05.

## Results

**Fig. 2:**
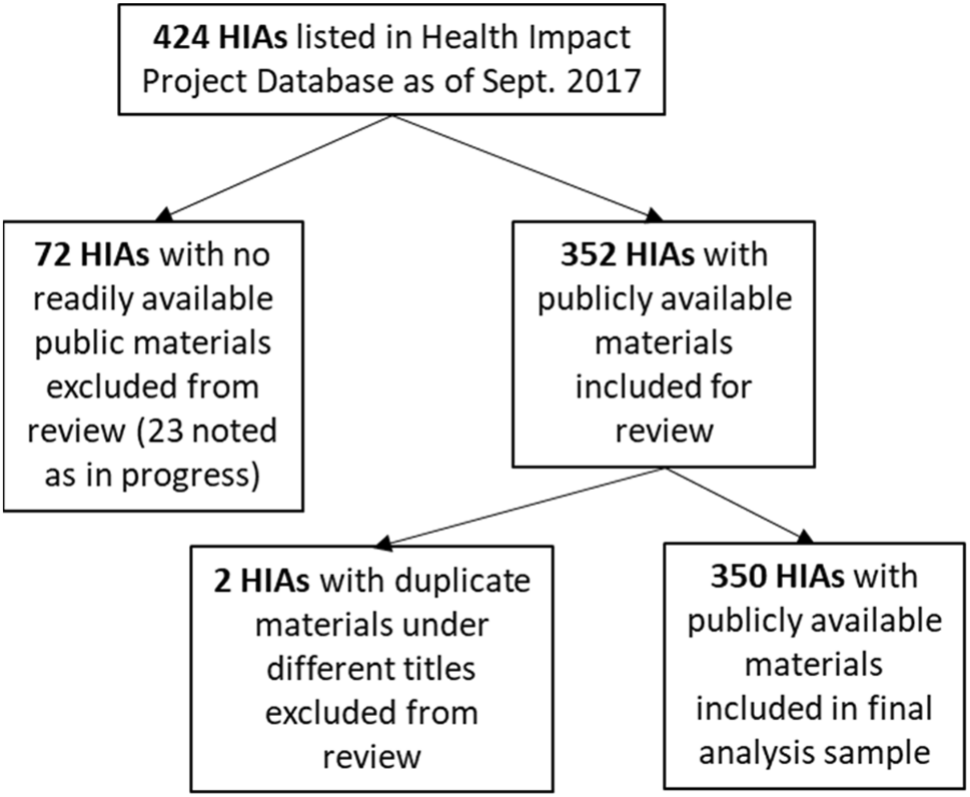
Inclusion diagram

Figure 2 illustrates how the 424 HIAs contained in the Health Impact Project database were narrowed to an analysis sample of 350 HIAs.

Research Question 1: To what extent have MCH-focused stakeholders been involved in HIA practice?

**Fig. 3:**
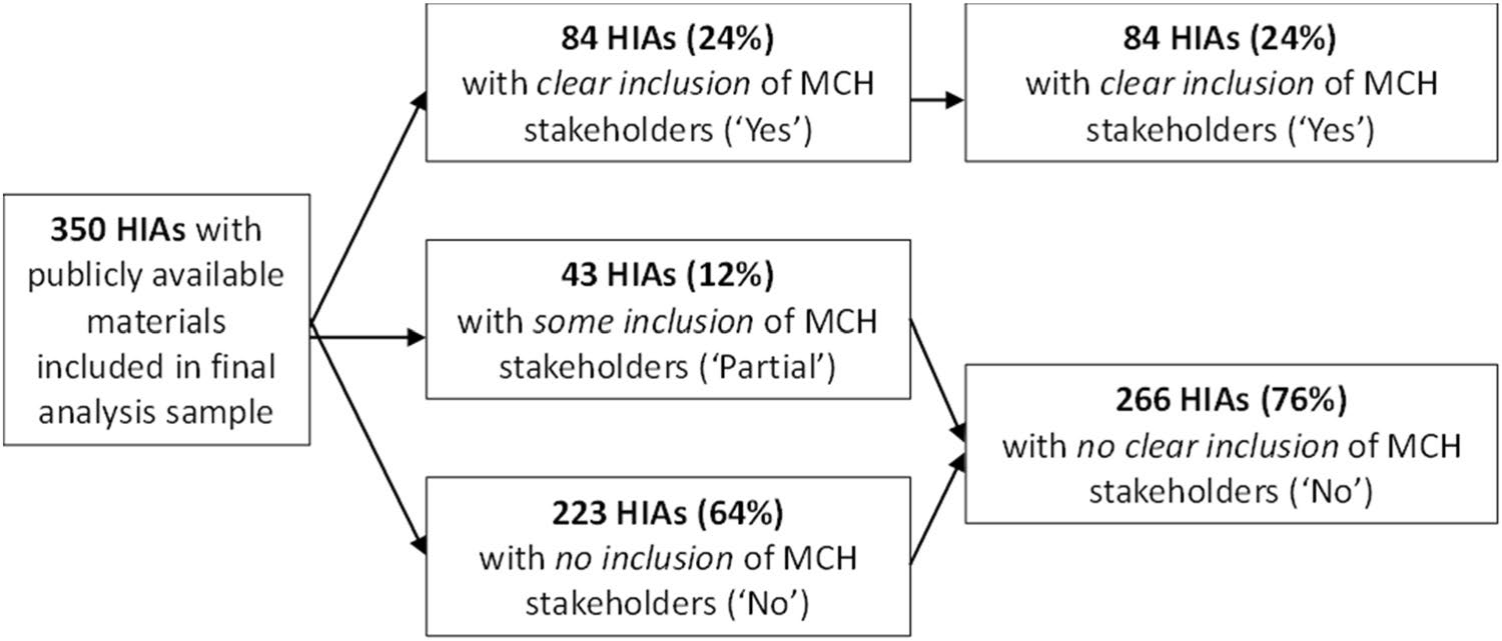
MCH stakeholder inclusion in HIAs

Slightly less than one quarter (24%) of the 350 HIAs reviewed demonstrated clear inclusion of MCH stakeholders (Fig. 3). The only example of an HIA that clearly indicated inclusion of a Title V agency considered impacts of potential changes to paid sick leave policy in Vermont (Vermont Department of Health, 2015). In that HIA, led by the Vermont Department of Health, representatives from the Division of Maternal and Child Health were listed as stakeholders, along with the Vermont Commission on Women, several school nurses, and a local child care facility. Under the definition of stakeholders described above, the most common stakeholder groups were school or other education system representatives and child or family-focused advocacy organizations, noted in 42% and 39% of HIAs coded as including MCH stakeholders, respectively.

Research Question 2: How often do and to what extent do HIAs examine MCH subpopulations?

**Fig. 4:**
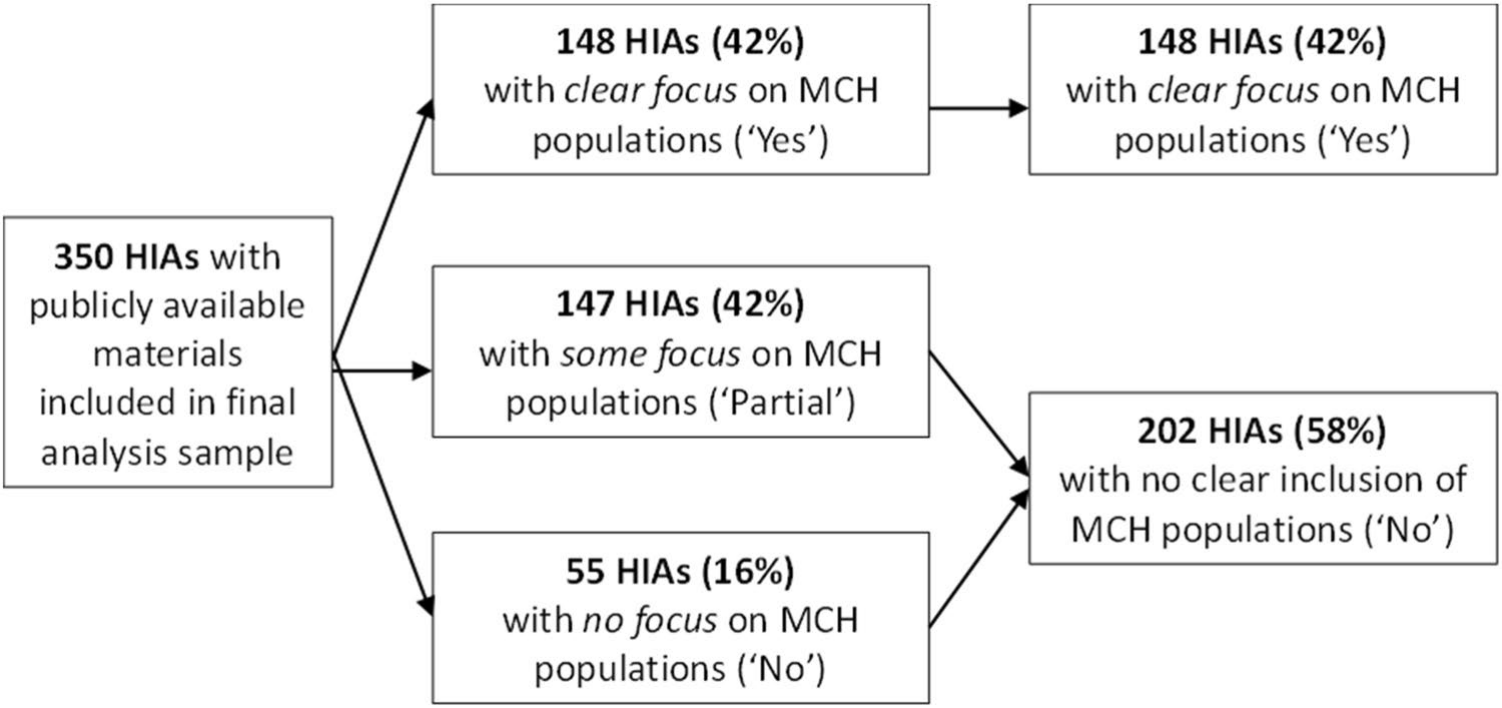
MCH population inclusion in HIAs

Eighty-four percent of the 350 HIAs reviewed at least partially considered MCH populations according to publicly available materials. As illustrated in Fig. 4, this 84% was split almost equally between HIAs designated as having a clear focus on MCH subpopulations (148 HIAs) and HIAs that only partially included them (147 HIAs).

Research Question 3: What proportion of HIAs include Title V National Performance Measures (NPMs) or closely related metrics in their analyses?

**Fig. 5:**
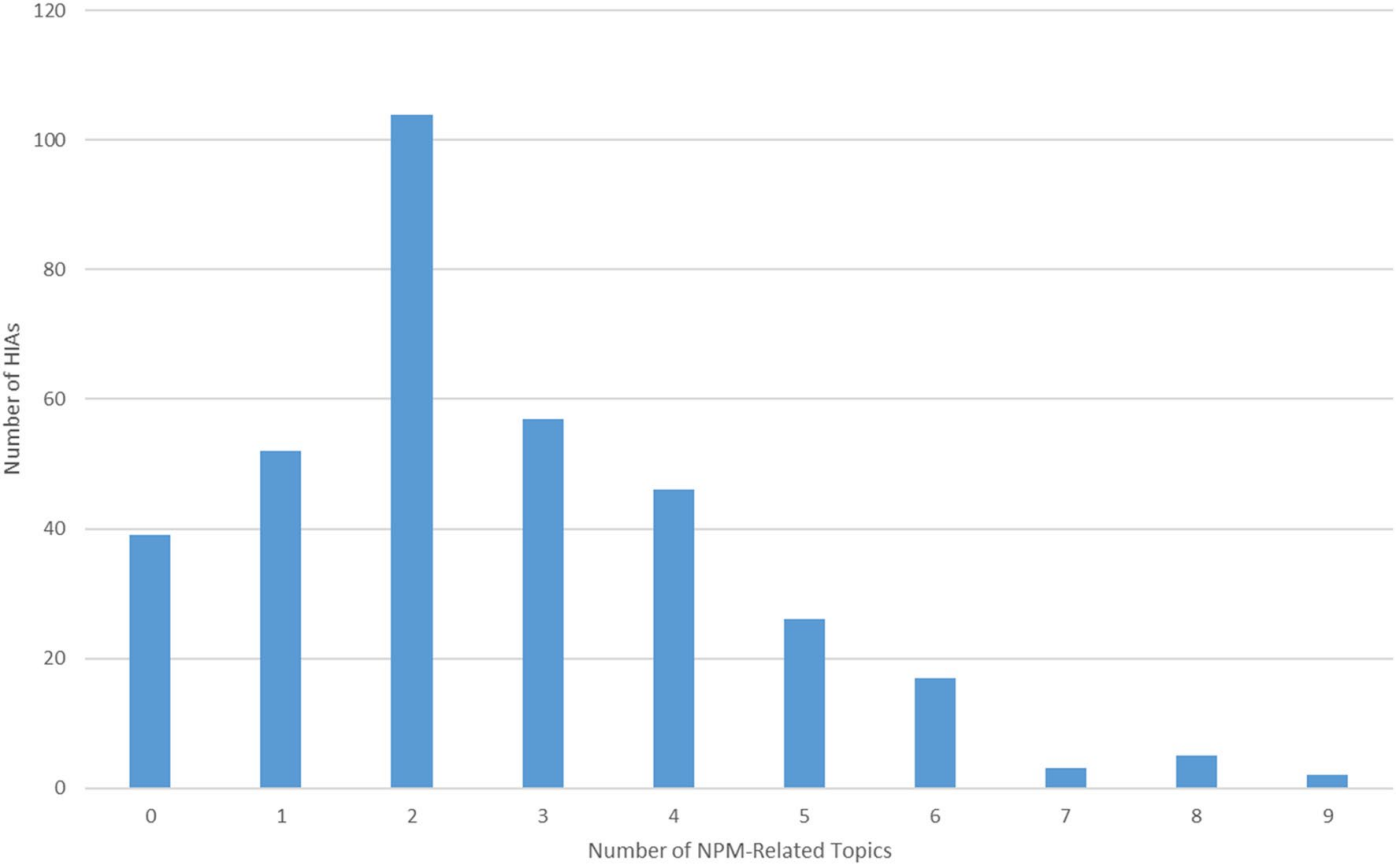
Frequency of HIAs by number of NPM-related topics included

**Fig. 6:**
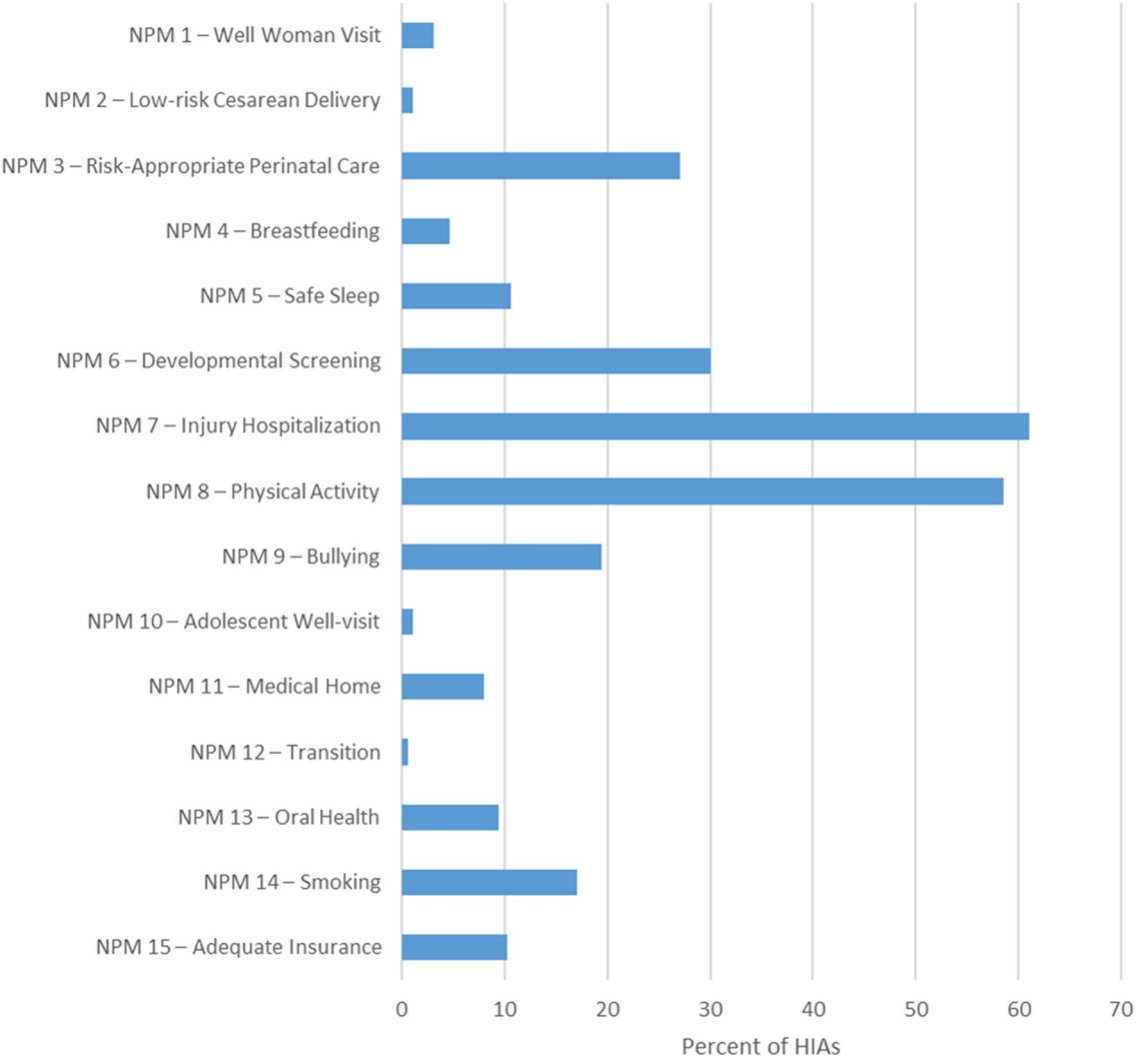
Percent of HIAs including each NPM-related topic

Eighty-nine percent of the 350 HIAs included information designated as closely related to or relevant for at least one NPM, and less than 2% included specific NPMs. The mean number of NPM-related topics included in an HIA was 2.62 (SD 1.80). The largest proportion of HIAs (30%) included two NPMs, and most (61%) included one to three topics (Fig. 5). All 15 NPMs were represented at least once across all HIAs reviewed (Fig. 6).

Injury and physical activity were the most common NPM-related topics, included in 61% and 59% of the 350 HIAs reviewed, respectively. None of the 214 HIAs including information about injuries used the specific NPM. However, all of them referenced injuries and hospitalizations in a way determined by reviewers to be relevant for MCH populations or practice. For physical activity, only one of the 205 HIAs coded as relevant for NPM 8 specifically included NPM itself.

Research Question 4: Are HIAs that engage MCH-focused stakeholders more likely to include information on MCH populations and NPM-relevant content?

The MCH stakeholder, MCH population, and NPM inclusion variables described above were used to test the hypothesis that HIAs involving MCH stakeholders are more likely to address both MCH populations and relevant measures. MCH stakeholder inclusion was the independent variable and dichotomized into HIAs with clear inclusion of MCH stakeholders (designated by reviewers as ‘yes’; 24% of the sample, 84 HIAs) and ones with no clear inclusion of MCH stakeholders (designated as either ‘partial’ or ‘no’; 76% of the sample, 266 HIAs). The dependent variable was a combination of MCH population focus and inclusion of NPM-related topics, where an HIA had to satisfy both conditions to be coded as ‘yes’ (41% of the sample, 143 HIAs).

HIAs that clearly included MCH stakeholders had seven times the odds of including both a focus on MCH populations and at least one NPM-relevant topic compared to HIAs that did not clearly include MCH stakeholders (OR 6.98; 95% CI 3.99, 12.20). This association is statistically significant (Chi-square, p < 0.001). Table 4 displays the distribution across these two variables.

**Table 4:** Inclusion of MCH stakeholders by MCH population focus and NPM-related topics

	HIAs with clear focus on MCH populations AND with NPM-related topics (% of total sample)	HIAs with no clear inclusion of MCH populations OR without NPM-related topics (% of total sample)
HIAs with clear inclusion of MCH stakeholders	63 (18%)	21 (6%)
HIAs with no clear inclusion of MCH stakeholders	80 (23%)	186 (53%)

To demonstrate the breadth of non-health policies informed by HIA, Table 5 includes the distribution of HIAs across different target sectors. Table 6 summarizes ten HIAs that illustrate the range of potential opportunities for MCH engagement in this type of cross-sector collaboration. These examples were selected to reflect variation in the three key review variables and in two key contextual factors: target sector and decision-making level (local, regional, state, or federal). They were also chosen to reflect the geographic diversity in HIA practice in the US.

**Table 5:** Distribution of HIAs by sector

Sector (as defined in source database)	Percent of all HIAs included in review (n = 350)	Percent of HIAs with clear MCH Stakeholder involvement (n = 84)
Agriculture, food and drug	8.6	15.5
Built environment	35.1	29.8
Education	4.0	13.1
Housing	7.7	6.0
Labor & Employment	4.6	7.1
Natural resources & energy	6.9	2.4
Transportation	21.4	14.3
Other*	11.7	11.8

*Includes the following sector designations, which each representing less than 10 HIAs in the sample: climate change, community development, criminal justice, economic policy, gambling, physical activity, and water

## Discussion

Nearly all HIAs reviewed (95%) considered an MCH population in some way or included information relevant to at least one NPM. In contrast, just over a third (36%) contained evidence of MCH stakeholder involvement. The current analysis demonstrates that MCH stakeholder involvement in an HIA is associated with greater odds of that HIA addressing MCH populations and relevant topics, suggesting an opportunity for MCH professionals and HIA practitioners to engage with one another in strategic efforts to inform decisions made outside the traditional public health sector. Aligning these efforts allows both MCH professionals and HIA practitioners to better advance a “Public Health 3.0” approach, wherein public health leaders partner across multiple sectors to address social, environmental, and economic conditions that affect health and health equity (DeSalvo et al., 2017).

MCH professionals have opportunities to advance health equity through cross-sector collaboration by actively pursuing involvement in HIA taking place in their respective jurisdictions and strategically considering it as a means for addressing issues relevant to their stakeholders and communities. Where HIAs are more common, there appears to be opportunity for intentionally integrating MCH perspectives into ongoing cross-sector collaborations addressing health determinants of significance for MCH outcomes. Where HIAs are less common, there may be opportunities for MCH leaders to initiate or strengthen those critical collaborations through the use of this specific tool. As practice continues to coalesce around advancing health equity, both in MCH efforts and through the HIA process, opportunities for collaboration will emerge and can be leveraged to advance population health outcomes for MCH populations. Collaborative cross-sector opportunities are not exclusive to HIA, and future research could expand upon the current review to further explore roles for MCH in other HiAP approaches.

HIA practitioners have opportunities to more actively seek engagement with MCH stakeholders, which could lead to broader applicability of HIA findings and recommendations. Incorporating more intentional MCH perspectives within HIA could be a mechanism to garner broader stakeholder and community engagement, which in turn can raise visibility of strategic approaches to population health and health equity. There could also be practical benefits in terms of access to and interpretation of MCH-relevant data that might otherwise be absent in a given assessment. Incorporating this perspective could strengthen the recommendations that are a primary output of the HIA process.

Although not coded as a key variable for this analysis, reviewers noted general MCH implications of recommendations in roughly two thirds of HIAs. If Title V or other MCH professionals are engaged in developing these recommendations, they can better ensure strategic relevance for MCH stakeholders and alignment with other MCH activities in a given jurisdiction. For example, with Title V involvement, an HIA could move past general discussions of an MCH-related issue to include NPM-specific data and strategies, resulting in direct advancement of relevant existing Title V priorities.

Intra-sector silos within public health may provide one explanation for underrepresentation of MCH stakeholders in HIA practice. When led by public health entities, HIAs are often housed within an environmental health or chronic disease unit; though in recent years, HIAs led by offices focused on evaluation, policy, or equity have become more common (J. Dills, personal communication, April 1, 2020). This assignment of HIA responsibility to specific public health content areas might raise barriers to supporting active involvement of MCH professionals from within an agency, among other bureaucratic challenges. Without active attempts to bridge these intra-sector silos and address these challenges, HIA work done by one arm of a public health agency can easily occur in isolation from the other. As MCH leaders become more engaged in upstream policy work, awareness of opportunities to leverage existing cross-sector experience from within their own agencies is an asset. HIAs potentially present this type of opportunity, as do HiAP approaches more broadly (Rudolph et al., 2013). A more thorough examination of variation in resources available to support HIA over time is beyond the scope of this review; however, future research could determine if greater availability of resources to support HIA is associated with greater likelihood of MCH inclusion in those HIAs.

In terms of connecting to sectors outside of public health, Table 5 shows the distribution of HIAs by target sector. HIAs aimed at informing decisions related to agriculture/food systems, education, and labor/employment have included MCH stakeholders more regularly than HIAs targeting other sectors, but all sectors have included MCH stakeholders to some degree. These data are included to demonstrate the breadth of sectors covered by HIAs generally and to note the sectors most common to HIAs that specifically include MCH stakeholders. This review does not consider the temporality of MCH involvement in HIA and the MCH-relevant content contained therein. Some decisions considered by HIAs may be more directly tied to MCH populations and have a readily apparent reason to include MCH practitioners, leading to more MCH-relevant content in those HIAs. For example, decisions made in the education sector are likely to influence children, so an HIA of those decisions may naturally include some level of MCH perspectives from the outset. As a collaborative approach, different perspectives inform HIAs throughout the process, with the screening and scoping stages as the most opportune time for robust MCH involvement that would potentially guide assessment and recommendations toward more MCH-relevant content.

### Limitations

Three interrelated limitations should be considered in interpreting these findings. First, approximately one in six HIAs listed in the Health Impact Project database did not have the publicly available documentation needed to be included in this analysis. Further, the database is populated mostly by voluntary self-reports from the HIA field, and thus, an unknown number of HIAs in the U.S. are not included in the data. How information from these HIAs might influence study results is unclear. Second, thoroughness of available HIA documentation varies widely (Rhodus et al., 2013). HIA is both a process and a report. This review only judged aspects of the process based on how well the associated reports (and other available documentation) described them. This limitation leads to an incomplete picture of HIAs that may have had a more robust process than reflected in their final reports and could have led to underestimating MCH stakeholder involvement. Finally, because of the exploratory nature of this review, the research team erred on the side of inclusivity when coding HIA content. Regular discussions of the data as they were being collected and resulting iterations of the review protocol may have introduced increased subjectivity in comparison to a more traditional systematic review of research studies. Future research examining involvement of MCH stakeholders in HIA practice could address some of these limitations by including interviews with leaders of and participants in specific HIAs.

### Conclusion

 HIA is an established tool that supports prospective engagement by public health stakeholders in non-health decision-making contexts. This exploratory review indicates limited involvement of MCH-focused practitioners in HIA practice to date. The results reveal that despite this lack of engagement, a large portion of HIA practice considers populations and measures with clear relevance to MCH. When the MCH workforce is involved, HIAs are significantly more likely to include specific MCH content. Therefore, intentional alignment of MCH and HIA practice, along with capacity building to support use of HIA by MCH stakeholders, would potentially reinforce strategies seeking to inform decisions made in other sectors. As noted earlier, these decisions influence social determinants of health, the inequitable distribution of which underpin many health disparities. HIA is one method through which public health perspectives can be integrated into these influential decisions for population health. Limited involvement of MCH practitioners in HIA points to a missed opportunity to ensure that their particular perspectives on public health are actionably included in these cross-sector collaborations to address social determinants.

**Table 6:** Selected HIA examples

HIA title, contextual data, and summary*	MCH Stakeholder involvement	MCH population focus	Summary and Selected examples of NPM-related Content	MCH-relevant recommendations	Summary of MCH Relevance from Review
Report Title: A Health Impact Assessment of a Food Tax in New Mexico Database Title: New Mexico Food Tax Year: 2015 Lead Organization: New Mexico Voices for Children Decision-making Level: State Target Sector: Economic Policy HIA Summary: New Mexico Voices for Children will conduct an HIA of a proposed tax on food in the state. In 2004, the New Mexico Legislature exempted food from the gross receipts tax, the state's version of the sales tax. However, state legislators "under pressure from local governments faced with declining revenues" are considering allowing localities to reinstate a tax on food Web link: http://www.nmvoices.org/wp-content/uploads/2015/11/HIA-report-full-web.pdf	Yes: New Mexico Voices for Children led this HIA and included several other MCH stakeholders from organizations such as the Department of Family and Community Medicine at the University of New Mexico Health Sciences Center; CHI St. Joseph’s Children (Home Visiting Program); and the UNM Department of Individual, Family, and Community Education: Nutrition/Dietetics Program and Department of Family and Community Medicine, and Pacific Institute for Research and Evaluation (PIRE)	Yes: Children and families, particularly lower income families, are an explicit focus of this HIA throughout. The report explores food insecurity and connections with child development, pregnancy outcomes, and the health of mothers, and includes implications for WIC and SNAP	Though it does not explicitly address any specific NPMs, this HIA is rich in MCH relevant content and includes information judged to be at least partially related to eight different NPMs (1, 3, 6, 8, 9, 11, 14, & 15). Sample content is included below specific to NPMs 6, 9, and 15 NPM 6—Developmental Screening (‘Partial’): Child development is referenced throughout the HIA as a key health outcome, but no specific screening data are mentioned. The summary of findings notes that the proposed food tax would be regressive and potentially “harm family economic security, which could have negative impacts on childhood development and learning capacity,” among other issues (p. 12). The report also notes that “taxing food could also have an adverse impact on food security, diet, and nutrition, which would have important and harmful implications for health, particularly…childhood development and learning capacity, malnutrition issues, the incidence of low birth-weight and/or preterm babies, and the need and demand for food assistance” programs (p. 12). The impact characterization table on p. 13 emphasizes that these negative impacts are most likely to burden “children and pregnant mothers in food insecure or low-income residents.” NPM 9—Bullying (‘Partial’): “Behavioral problems at school” is included as an intermediate impact in the pathway that considers how increased food costs could potentially connect to health outcomes of concern, such as “cognitive, development and educational outcomes” or “diet- and nutrition-related disease (diabetes, hypertension, heart disease, stroke, obesity)” (p. 26). While not stated explicitly, these behavioral problems could include bullying NPM 15—Adequate Insurance (‘Partial’): In details of the HIA analysis of food tax impacts on food security, diet, nutrition, and health, some baseline statistics about insurance coverage for children in the state are included: “The state ranks poorly (40th nationwide) in the percentage of children without health insurance (7% in NM versus 6% in the U.S.), which impacts access to and usage of preventive services as well as treatments needed for a range of conditions for children. However, since expanding Medicaid, New Mexico has seen a dramatic drop in the number of uninsured children. The number fell from 14% in 2008 to 9% in 2013, then to 7% in 2014” (p. 35). While these statistics do not directly correspond to the NPM “Percent of children ages 0 through 17 who are adequately insured,” they are clearly relevant for the topic of insurance coverage. In the HIA, this topic is part of the context in which potential impacts of the food tax on household economics are explored	The HIA puts its recommendations in the context of a “key finding…that the tax code is an important health determinant and can play a significant role in child and family health and well-being,” adding that “policy recommendations…are driven by that finding and the idea that changes to tax code should improve the health and well-being of New Mexico families” (p. 46). The report includes several recommendations that would likely be of particular relevance for MCH stakeholders in the state, for example “Increase current state tax credits and create new credits for low-income families” and “Increase and/or maximize programs that help to improve food access and diet- and nutrition-related health outcomes of vulnerable populations” (pp. 46–47)	High: This HIA was led by and engaged multiple MCH stakeholders throughout the process. The report has a clear focus on potential impacts for children, pregnant individuals, and families. Content related to eight NPMs is presented, including well-woman visits, risk-appropriate perinatal care, developmental screening, physical activity, bullying, medical home, smoking, and adequate insurance. The recommendations are specifically placed in the context of improving health determinants for MCH populations, and several would be particularly relevant for MCH stakeholders in New Mexico
Report Title: A Health Impact Assessment of the Healthy Families Act of 2009 Database Title: Federal Paid Sick Days Year: 2009 Lead Organizations: Human Impact Partners and San Francisco Department of Public Health Decision-making Level: Federal Target Sector: Labor and Employment HIA Summary: An HIA that addressed health implications of the federal Healthy Families Act of 2009, a bill that would entitle all employees to accrue paid sick time at a rate of 1 h of paid sick time for every 30 h worked, up to nine days per year Web link: http://www.pewtrusts.org/~/media/assets/2009/09/federalpaidsickdays.pdf?la=en	Yes: This HIA clearly included MCH-related stakeholders. The National Partnership for Women & Families commissioned this HIA. Representatives from the Labor Project for Working Families, the Institute for Women’s Policy Research, the National Partnership for Women & Families, the Maine Women’s Lobby, and the Maine Women’s Policy Center all participated in the HIA Advisory Committee and served as report reviewers	Yes: This HIA most directly considers MCH populations in respect to children as dependents of workers, especially mothers, and their ability to take time off work to care for them when needed	This HIA includes reference to NPM 1 and contains information judged to be at least partially related to three other NPMs (3, 11, & 15). Sample content is included below specific to all four NPM 1—Well-Woman Visit (‘Yes’): The assessment found that paid sick days was a statistically significant predictor of medical visits, including primary care visits. National Health Interview Survey (NHIS) data on visits to an OB/GYN were used in the analysis and are noted specifically in a description of the analysis sample: "Among women, close to half (47.7%) indicated that they had visited an obstetrician or gynecologist in the past year." (p. 62) NPM 3—Risk-Appropriate Perinatal Care (‘Partial’): In discussing effects of paid sick days on wage loss, the HIA notes that “people with lower incomes have higher risks than people with higher incomes for giving birth to low birth weight babies.” (p. 40) LBW is also included in a table of preventable hospitalization rates in the US (p. 29) NPM 11—Medical Home (‘Partial’): While not explicitly identifying the proportion of children with a medical home, the following is noted among key findings: “a parent’s ability to take paid leave leads to better physical and emotional health outcomes for children with special needs (p. 5).” This content is inclusive of the NPM11 consideration of children with special health care needs having a medical home NPM 15—Adequate Insurance (‘Partial’): In a section discussing ‘Care for Dependents among Workers With and Without Paid Sick Days,’ the report notes data showing that “paid sick days was not statistically associated with delayed or no care for family members among those without health insurance; however, paid sick days significantly decreased the experience of delayed care among those with health insurance. This indicates that paid sick days may further facilitate access to health care for dependents of workers who have health insurance (p. 25).”	This HIA report has no list of specific recommendations, likely due to contextual factors beyond the scope of this review; however, it does emphasize the overarching finding “that the best available public health evidence demonstrates that the Healthy Families Act of 2009 would have significant and beneficial public health impacts (p. 7).” Of particular relevance from an MCH perspective is additional emphasis that the evidence reviewed “was consistent with the hypothesis that a requirement for paid sick days would protect and enable worker health [and] worker care for sick dependents (p. 5),” who in many cases are children	High: Documentation indicates multiple MCH stakeholders engaged in this HIA, and the analysis clearly describes impacts on mothers, families, and children. Several NPM-relevant topics are covered, including well-woman visit, risk-appropriate perinatal care, medical home, and adequate insurance. The publicly available HIA report does not explicitly make recommendations, but it does emphasize the potential for positive health impacts based on a detailed accounting of assessment findings
Report Title: A Health Impact Assessment of the 2015 Qualified Allocation Plan for Low-Income Housing Tax Credits in Georgia Database Title: Georgia Qualified Allocation Plan for Low-Income Housing Tax Credits HIA Year: 2015 Lead Organizations: Georgia Health Policy Center Decision-making Level: State Target Sector: Community Development HIA Summary: This HIA will inform the 2015 Georgia Qualified Allocation Plan for low-income housing tax credits by assessing the proposed criteria for allocating the credits and considering how these criteria will impact health through the effects on housing for vulnerable populations and community development decisions Web link: http://ghpc.gsu.edu/download/an-hia-of-the-2015-qualified-allocation-plan-for-low-income-housing-tax-credits-in-georgia/	No: The HIA summary report does not indicate the involvement of any MCH-specific stakeholders. Both the state Department of Education and Department of Early Care and Learning appear to have been consulted as part of the analysis, but the extent of their involvement is unclear in available documentation, and neither was represented on the HIA Steering Committee	Yes: One of the three major topics in the scope of this HIA is how to encourage access to educational opportunities through affordable housing tax credit allocations, which is tied directly to child health. Children and families are also noted as a population of interest generally throughout the HIA. Documentation does not explicitly reference mothers or women’s health	Though it does not explicitly address any specific NPMs, this HIA has content judged to be at least partially related to four (3, 6, 7, & 14). Sample content is included below for NPMs 6, 7, & 14 NPM 6—Developmental Screening (‘Partial’): Implications of housing location and design on childhood development are noted a few times in the HIA, most specifically in relation to encouraging access to educational opportunity. In the evidence review for that section, the report notes “early childhood experiences—from zero to five years, and even before birth—have been identified as crucial developmental factors that can determine lifelong outcomes” (p. 19). Though this topic appears to be the foundation for a significant part of the HIA, specific data corresponding to measures of child development or screening rates are not included NPM 7—Injury Hospitalization (‘Partial’): Injury risks are noted throughout and referenced in this summary statement from the report abstract: “A fully funded affordable housing program that is tuned to reduce injury and illness could improve wellbeing, increase productivity, and reduce health care costs in Georgia” (p.ii). Within the HIA, youth injuries are most directly referenced in a table entitled “Sample Content from the Desktop Assessment of Healthy Community Design,” which notes that “the home environment can contain many injury hazards, and the lower income populations served by [tax credit] developments are especially vulnerable,” adding the perspective that " Unintentional injuries (excluding falls and car crashes) are the leading cause of emergency room (ER) visits for Georgians between the ages of 1 and 19 " (p. 28) NPM 14—Smoking (‘Partial’): In the rationale for a recommendation to develop smoke-free housing, the HIA states that “tobacco use, particularly smoking, is proven to be the most prevalent underlying cause of death in the US. In addition to increasing risk of death, disability, and high costs for the smoker, habitual smoking also leads to negative health outcomes for other members of the household and community that are exposed to environmental tobacco smoke” (p. 24). Though no specific statistics are presented for women who smoke during pregnancy or children who live in households where someone smokes, this content was judged to be generally relevant for this NPM because it could have direct bearing on both	This HIA makes several recommendations with relevance for MCH. Potentially the most relevant for improving child health through affordable housing policy are recommendations to develop scoring incentives for “locating in the attendance zones of above average schools” and “near high quality early learning facilities” (p. 3). Other recommendations with direct bearing on MCH stakeholders and/or populations are summarized in a table of “Sample Content from the Desktop Assessment of Healthy Community Design” (pp. 24–25) and include locating away from busy roadways and other sources of air pollution, implementing smoke free housing policies, and promoting health programming on-site at housing development once placed in service	Moderate: Though this HIA did not directly include MCH stakeholders, some clear opportunities exist for them to be involved in implementing recommendations and/or furthering the alignment of affordable housing policy and public health, particularly for mothers, children, and families. Children are noted throughout as a population particularly susceptible to impacts of housing policies, and several NPM-relevant topics are covered, including risk-appropriate care, developmental screening, injuries, and smoking. Recommendations are primarily geared toward affordable housing developers and policy-makers; however, some clear opportunities exist for MCH stakeholders to become more involved in affordable housing policy in Georgia
Report Title: Community Development and Health: A Health Impact Assessment to Inform the Community Investment Tax Credit Program Database Title: Massachusetts Community Investment Tax Credit Grant Program HIA Year: 2014 Lead Organizations: Health Resources in Action, in partnership with the Massachusetts Department of Public Health and the Metropolitan Area Planning Commission Decision-making Level: State Target Sector: Community Development HIA Summary: This HIA to inform the promulgation of regulations by the Massachusetts Department of Housing and Community Development will guide the release of funding for community development corporations Web link: http://www.pewtrusts.org/~/media/assets/external-sites/health-impact-project/hria-mapc-citc-report.pdf?la=en	No: The HIA report does not explicitly note the involvement of any MCH stakeholders. Contributing authors came from the Bureau of Community Health and Prevention and the Bureau of Environmental Health at the Massachusetts Department of Public Health (MDPH), and from Health Resources in Action. These contributing authors from the public health perspective may have incorporated some undocumented involvement of MCH perspectives. Broad stakeholder engagement is noted throughout without directly identifying who those stakeholders were, so some were possibly MCH-related	Partial: Children from low-income families are identified as one of “the vulnerable populations that CDCs [Community Development Corporations] directly impact through their work” (p. 77), and they are noted throughout as particularly vulnerable to the effects of poverty, one of the main health determinants examined in the HIA. The assessment also notes “more low income households with young children (43%) in the areas served by CDCs as compared to areas not served by CDCs (29%),” so children are certainly considered broadly; however, the analyses and recommendations presented do not appear to explicitly target children (p. 36). Mothers and women are not an identified population of focus in the HIA	Though it does not explicitly address any specific NPMs, this HIA has content judged to be at least partially related to five (6, 8, 9, 11, & 14). Sample content is included below for NPMs 6, 11, & 14 NPM 6—Developmental Screening (‘Partial’): In discussing the role of CDCs in promoting Asset Development, which includes “activities that improve an individual’s ability to acquire and maintain assets,” the HIA includes passing reference to developmental delays in children and specifically connects housing insecurity to “behavioral problems, developmental delays, and poor mental health among children” (p. 66). By potentially facilitating CDC activities related to Asset Development, the proposed Community Investment Tax Credit (CITC) program could help reduce these issues. No data about screening rates are included or referenced NPM 11—Medical Home (‘Partial’): While not specifying access to a medical home for children with or without special health care needs, the HIA does include access to medical care as one of the health outcomes likely to be positively impacted by the CITC program through all pathways examined. For example, local economic development activities facilitated by the program “can contribute to increases in residents’ income, which enables individuals and families to afford…medical care” (p. 17). For the purposes of this review, this increase in access is assumed to precipitate improvement in this NPM NPM14—Smoking (‘Partial’): The HIA makes a few connections between CDC activities facilitated by the CITC program and reducing unhealthy behaviors, including smoking. In discussing the broad implications of local economic development, the report notes that “the creation and sustainability of small businesses in a community” can minimize neighborhood deprivation that “is associated with increased risk of physical inactivity, unhealthy diet, smoking, and obesity” (p. 63). They also briefly discuss implications of youth empowerment programs that “may impact a wide range of health behaviors and outcomes as well as help to reduce risky and unhealthy behaviors, such as smoking… and promote more positive behaviors in this group” (p. 73). While the HIA does not directly addresses women who smoke during pregnancy or children who live in households where someone smokes, this content was judged to be generally relevant for this NPM because it could have direct bearing on both	Recommendations do not directly address MCH topics, stakeholders, or population to a substantial degree; however, because many call for better integration of health perspectives into community development activities, they may present an opportunity for more targeted MCH involvement. For example, a recommendation made to the CITC implementing agency is to “Revise Future Notices of Funding Availability (NOFA)—Community Investment Plans” (CIP) requirements to “include…a prompt for health related data as part of characterizing constituencies to be served” and to “identify…health care and public health organizations as suggested stakeholders” (p. 99). MCH perspectives could be part of providing those data and be identified among those stakeholders. Several other recommendations could similarly benefit from involvement of MCH stakeholders as part of a general increase in alignment of community development and public health activities across Massachusetts encouraged by this HIA	Moderate: Documentation does not indicate MCH stakeholder involvement in this HIA, but it does reference children as a vulnerable population that could be impacted positively by implementation of the CITC program. The report offers some content at least partially relevant for five NPMs, including developmental screenings, physical activity, bullying, medical home, and smoking; however, none of these topics is thoroughly examined. Generally, “this HIA predicts that the CITC will have an overall positive impact on public health of low- and moderate-income households across the state served by the certified CDCs” (p. 95), and recommendations set a context in which MCH could clearly become a more integral part of the of community development programs, plans, and policies
Report Title: Full-Day Kindergarten in Nevada: A Health Impact Assessment Database Title: Full-Day Kindergarten Year: 2015 Lead Organizations: University of Nevada, Las Vegas Decision-making Level: State Target Sector: Education HIA Summary: The University of Nevada, Las Vegas conducted an HIA to inform decisions by the Nevada State Legislature on proposed modifications to the availability of full-day kindergarten and on allocating state funding to support full-day kindergarten in high-risk schools Web link: http://www.pewtrusts.org/~/media/assets/external-sites/health-impact-project/unlv-2015-full-day-kindergarten-in-nv-report.pdf?la=en	Yes: MCH stakeholders, mainly from the education sector, were involved in this HIA as part of its Steering Committee. Organizations represented include several elementary schools, education advocacy groups, and the Children’s Advocacy Alliance. The lead authors were from the UNLV School of Community Health Sciences, and at least one local health department was also included on the Steering Committee	Yes: This HIA considers impacts of kindergarten availability on three main health determinants for children: educational attainment, physical development; and access to school-based services. The HIA does not consider maternal health in any detail	This HIA includes reference to NPMs 6 & 11 and contains information judged to be at least partially related to six others (3, 7, 8, 13, 14, & 15). Sample content is included below specific to NPMs 6, 8, 11, & 13 NPM 6—Developmental Screening (‘Yes’): An overarching finding of this HIA is that “by increasing time spent in school, access to [Full-day Kindergarten] FDK could…improve physical development through school-based nutrition education and physical activity” (p. 9). A table of “Measures for Health of Children in Nevada” shows that 21.9% “of children age 10 months to 5 years received a standardized screening for developmental or behavioral problems,” compared to 30.8% nationally (p. 84). Though the statistic does not explicitly state that these data reference use of a parent-completed screening tool, reviewers considered this information as corresponding to this NPM. Though this specific measure is included, it does not appear to have been a driving component of the assessment NPM 8—Physical Activity (‘Partial’): This topic is a major focus of the HIA, which, among other high-level findings, notes that “school-based physical activity is effective in increasing overall levels of physical activity” for children (p. 12). Increasing access to FDK would potentially increase access to school-based physical activity. While the HIA does not state percentages of children who obtain the recommended amount of physical activity by age group as specified by the NPM, it does include information about how many minutes of physical education and recess are offered in schools per week (p. 79) NPM 11—Medical Home (‘Yes’): A table of “Measures for Health of Children in Nevada” shows that 44.6% of children in the state “receive care within a medical home,” compared with 54.4% nationally (p. 84). Because this topic was only found in eight of the 350 HIAs included in this review, this statistic was considered to correspond to the NPM, despite not mentioning children with special health care needs. Though included in this baseline table, this topic is not included anywhere else in the analysis, and children with special needs are not mentioned in any actionable way at all NPM 13—Preventive Dentist Visit (‘Partial’): Similar to the developmental screening and medical home NPMs, oral health is referenced in the HIA, but only as part of the general baseline table of “Measures for Health of Children in Nevada,” which notes 67.4% “of children with preventive dental visit in the last year,” compared to 77.2% nationally (p. 84). This content was considered as partial inclusion of the NPM because it did not specify the age range, nor did the HIA include information about pregnant individuals’ dental visits. This topic is not represented anywhere else in the HIA	Because they consider FDK, all the recommendations have some level of relevance for MCH, as they deal with improving health of children through various pathways. An example particularly relevant for potential collaboration with MCH stakeholders outside the education system is that “school districts, the Nevada Division of Public and Behavioral Health, and local health departments could consider collaborating to measure height and weight annually and to track data over time by using unique student identification numbers to maintain the confidentiality of personally identifiable information and make the data publicly available for monitoring purposes” (p. 11)	High: This HIA included several MCH stakeholders from the education sector and a few from public health. By nature of considering an education topic, child health is clearly an associated focus. The report presents content related to eight NPMs, including risk-appropriate perinatal care, developmental screening, injury, physical activity, medical home, dental visits, smoking, and adequate insurance. However, many of these are only briefly mentioned and do not receive any attention in the assessment or findings. Recommendations are for expanding and improving FDK in Nevada and offer several opportunities for further MCH engagement around nutrition, education, health, and school-based services
Report Title: Health Impact Assessment of a Cap‐and‐Trade Framework Database Title: California Cap and Trade Rulemaking Year: 2010 Lead Organizations: California Department of Public Health and California Public Health Institute Decision-making Level: State Target Sector: Climate Change HIA Summary: An HIA to inform the development of new carbon cap and trade regulations (Assembly Bill 32) by the California Air Resources Board Web link: http://www.pewtrusts.org/~/media/assets/2010/12/01/californiacapandtrade.pdf	No: The publicly available report does not clearly describe involvement of MCH stakeholders. The impetus for the HIA was a public meeting of the Climate Action Team Public Health Workgroup (CAT PHWG), hosted by the California Department of Public Health (CDPH) and the California Air Resources Board (ARB), who “would co‐lead the HIA of cap-and-trade, using expertise and resources from both agencies to perform the assessment” (p. 17). Stakeholder input is referenced throughout, but only CDPH, ARB, and CAT PHWG are explicitly identified. Documentation is unclear on if MCH perspectives were intentionally engaged, either from within CDPH or from other organizations	Yes: Young children are explicitly mentioned as a population of focus with existing health vulnerabilities, and women are mentioned several times in the context of pregnancy	Though it does not explicitly address any specific NPMs, this HIA is has content judged to be at least partially related to nine NPMs (1, 3, 4, 6, 7, 8, 9, 14, & 15). Sample content is included below specific to NPMs 3, 4, and 7 NPM 3—Risk-Appropriate Perinatal Care (‘Partial’): The assessment includes low birth weight as a community health indicator, and a profile of an environmental justice community in Los Angeles County notes that neither the neighborhood nor the county “meets the low birth weight target of 5% (Target 16-10a) set by Healthy People 2010,” adding that “exposures to air pollution during pregnancy have been shown to have detrimental effects on preterm birth, fetal growth and development, and birth weight; and risk for low birth weight may be compounded by excess social and environmental stress in environmental justice communities (p. 66).” Though this content does not consider the NPM criteria of care for very low birthweight births, it does consider environmental conditions that could lead to low or very low birthweight and was therefore noted as relevant to this review NPM 4—Breastfeeding (‘Partial’): Breastfeeding is noted as one of many evidence-based strategies for improving community health on which cap-and-trade revenues could be spent. Specifically, “support breastfeeding through policy change and maternity care practice” is included in a table of interventions in Appendix B (p. 112). No data or other evidence regarding breastfeeding are offered elsewhere in the report NPM 7—Injury (‘Partial’): Injuries are noted in discussions of climate-related environmental hazards like wildfires (p. 10) and as part of general statements characterizing community vulnerabilities ‘to illness and injury’ (p. 98). Safety is also mentioned in the context of traffic-related injuries (p. 11) and violence (p. 98). Unintentional injuries are included in a table of leading causes of mortality in one of the case study communities (p. 74), but injury hospitalizations are not mentioned specifically. The only place where youth injuries are explicitly noted is the discussion of using cap-and-trade revenues to fund community health interventions, which states, “effective violence prevention programs reduce injuries and deaths, especially in young people of color, provide safer environments for walking for physical activity, and increases investments in disadvantaged neighborhoods” (p. 104)	The HIA makes recommendations specific to the cap-and-trade program and for the use of proceeds it would generate. From the program-specific set, some of the most MCH-relevant consider employment and labor transition mitigations, wherein “investments in worker transition assistance, adult education, and temporary insurance... reduce the economic dislocation and related adverse health impacts that are related to unemployment and job insecurity, including health insurance gaps,” which would include coverage for women and dependent children (p. 96). From the recommendations on use of cap-and-trade proceeds, the HIA notes support for “the allocation of a significant portion of revenues to improve the health of vulnerable and disadvantaged communities,” which explicitly includes children (p. 97)	Moderate: Although the HIA report does not indicate the intentional involvement of MCH stakeholders, much of the content has relevance for women, families, and children, the latter of which is considered to be a particularly vulnerable population within the assessment. The broad scope of impacts considered in the HIA lends itself to the inclusion of numerous topics that are at least partially relevant to nine NPMs, including well-woman visits, risk-appropriate perinatal care, breastfeeding, developmental screening, injuries, physical activity, bullying, smoking, and adequate insurance. Recommendations are similarly broad, but the most MCH relevance stems from a proposed process for using cap-and-trade revenues to support community health interventions, many of which would have a more direct relationship with MCH stakeholder priorities than what is reflected in this HIA
Report Title: Health Impact Assessment: National Nutrition Standards for Snack and a la Carte Foods and Beverages Database Title: Same as above Year: 2012 Lead Organizations: The Kids Safe & Healthful Foods Project and the Health Impact Project, both collaborations of The Pew Charitable Trusts and the Robert Wood Johnson Foundation, working in collaboration with Upstream Public Health Decision-making Level: Federal Target Sector: Agriculture, Food and Drug HIA Summary: An HIA to inform USDA as it updates nutrition standards for foods and beverages that are sold outside of the school meal programs, and to better understand how standards might affect student health and school finances Web link: http://www.pewtrusts.org/~/media/assets/2012/06/snackfoodshealthimpactassessment.pdf	Yes: MCH stakeholders involved in this HIA primarily focus on children in the school setting and appear to have had a substantial role in the execution of the project. They included The Kids’ Safe and Healthful Foods Project, which staffed the HIA research team, and representatives from the Division of Adolescent and School Health at the Centers for Disease Control and Prevention and the Center for Safe and Healthy Schools at the National Association of State Boards of Education, who served on the HIA advisory committee. “The process also required extensive interviews and involvement of a wide array of experts and stakeholders from academia, industry, the public health community, and those individuals most affected at the ground level, such as teachers, students, and parents, in planning, researching, and peer reviewing the study” (p. 2)	Yes: This HIA focuses on school-aged children and adolescents in the United States. More specifically, it “considers several key research questions related to school food services, diet and nutrition, and vulnerable populations (including low income and ethnic minority students)" (p. 3). These research questions specifically focus on how national policy change could lead to changes in “children’s health” (p. 17). Documentation indicates no explicit focus on maternal health within this HIA	This HIA includes reference to NPM 8 and contains information judged to be at least partially related to three other NPMs (3, 9, & 13). Sample content is included below specific to all four NPM 3—Risk-Appropriate Perinatal Care (‘Partial’): Low birth weight is noted in a table summarizing "effects of childhood food insecurity" (p. 26). The HIA does not return to this topic in any substantive way within the analysis or recommendations NPM 8—Physical Activity (‘Yes’): A key research question for this HIA is “If revenue changes occur [because of the policy changes being considered], will they affect student health via changes to enrichment learning opportunities and school-supported physical activity?” (p. 17). In describing baseline conditions in respect to child physical activity the assessment references data from the 2003–2004 NHANES showing that “nearly half (42%) of children between six and 11 obtain 60 min a day of physical activity, while less than one-tenth (8%) of adolescents reach this level” (p. 39). This content corresponds to the NPM 8 statistic, but the HIA was unable to find sufficient evidence linking a national snack and a la carte foods and beverage rule to school physical activity programs (p. 54) NPM 9 -Bullying (‘Partial’): This topic is touched upon a couple of times in general discussions of negative implications of childhood obesity. For example, “Childhood obesity can reduce children’s focus through poor body image or depression, or as a result of bullying” (p. 72) NPM 13—Preventive Dental Visit (‘Partial’): While the HIA does not consider dental visits specifically, it does include dental cavities among “secondary, indirect outcomes related to the policy,” and it reports a possible reduction in risk for some children with a national snack and a la carte food and beverage rule (p. 70). The assessment also ties oral health to learning potential: “Dental decay and oral health problems also place children at increased risk of poor learning outcomes and, if untreated, can lead to other chronic illnesses. Low-income children are disproportionately affected by tooth decay, particularly untreated cavities. Studies show that children with tooth decay are absent from school more than their peers and, when present, are often in pain and unable to focus. Dental decay can exacerbate problems for children who may already be at educational risk, contributing to difficulty learning” (p. 72)	The HIA offers detailed recommendations that are summarized thusly, “USDA should promulgate scientifically sound nutrition standards and adopt practices—as recommended by this HIA—that are most likely to maximize positive health impacts while assisting schools in effectively implementing new standards” (p. 98). The detailed recommendations are all clearly relevant for children’s health, and one with potentially the most relevance for MCH stakeholders is for USDA to “collaborate with states and non-governmental organizations to monitor the implementation of the standards” (p. 87)	High: This HIA examines potential impacts on children’s health associated with a potential USDA policy to implement national nutrition standards for snack and a la carte foods and beverages sold in schools. Several MCH stakeholders were involved in producing the HIA, and the report considers topics that are relevant for at least four NPMs, including risk-appropriate perinatal care, physical activity, bullying, and dental visits. Recommendations are geared specifically toward USDA policy, but because of their potential impact on children opportunities for MCH to be involved in their implementation are apparent
Report Title: Healthy Waterways: A Health Impact Assessment of the City of Rochester, New York’s Local Waterfront Revitalization Program Database Title: Rochester Waterfront Revitalization Plan Year: 2013 Lead Organizations: University of Rochester Decision-making Level: Local Target Sector: Built Environment HIA Summary: An HIA to help inform a waterfront revitalization plan in Rochester, New York Web link: http://www.pewtrusts.org/~/media/assets/2013/05/01/rochesterhiareport1.pdf?la=en	Partial: The HIA report identifies at least one MCH stakeholder organization by name: Healthi Kids, Finger Lakes Health Systems Agency (currently Common Ground Health). The extent of their involvement in the HIA process is unclear, but as a named stakeholder in the report they are assumed to have had a role throughout the process. The HIA was led by the University of Rochester Medical Center Department of Environmental Medicine, which may have also included an MCH perspective	Yes: Children are identified as one of the three main populations of concern in this HIA, which examined the Local Waterfront Revitalization Program (LWRP) in Rochester and its potential to influence community health. Maternal health is not included in this HIA; though it does reference maternal education	Though it does not explicitly address any specific NPMs, this HIA has content judged to be at least partially related to four (3, 7, 8, & 14). Sample content is included below for NMPs 3, 7, & 8 NPM 3—Risk-Appropriate Perinatal Care (‘Partial’): Though it does not consider perinatal care specifically, the HIA does consider how low and very low birthweight are related to “environmental burdens on vulnerable populations” in specific neighborhoods (p. 33). “Concerns about children’s environmental health extend to all children who use waterfront resources (such as beaches, parks, and fishing areas), but those who live in waterfront neighborhoods are most likely to be affected by changes resulting from the LWRP” (p. 33) NPM 7—Injury Hospitalization (‘Partial’): “Physical injury” is among the health outcomes included in the scope of the assessment, and physical safety is among the health determinants for which LWRP impacts are considered (p. 20). “Concerns about physical safety include unintentional injuries such as accidents and drowning, as well as injuries related to crime. LWRP-related changes in the physical environment may change physical safety hazards, use patterns that expose people to risk, and perceptions of safety” (p. 22). The HIA goes on to note that “unintentional injury is a significant cause of hospitalization and mortality” and “is the leading cause of death for New Yorkers between the ages of 1 and 44” (p. 44). No detailed statistics about youth injury hospitalizations are included NPM 8—Physical Activity (‘Partial’): Physical activity as a helth determinant is predicted in the HIA to be influenced by all “five types of waterfront changes addressed in the LWRP: waterfront trails, beach redevelopment and management, built environment, water-based recreation, and stormwater management” (pp. 1–2). A few details are specific to physical activity children, for example, it “has been shown to improve mental health and improve children’s performance in school" (p. 52). Though children are identified as a vulnerable population, the HIA does not include a robust characterization of data about physical activity rates for children	This HIA has few directly MCH relevant recommendations, but some of the overarching recommendations about explicitly including community health in the goals and vision of the LWRP could have broad relevance for MCH stakeholders. A few detailed recommendations would specifically improve conditions for children, including integrating “trails into Safe Routes to School programs" and "coordinating with the Rochester City School District and community partners to develop programs that highlight historical, cultural, and environmental resources along the trails, encourage physical activity, and educate about safe trail use" (p. 114)	Low: This HIA included at least one MCH stakeholder in the process, and it identifies children as a particularly vulnerable population. The analysis contains some content partially relevant to four NPMs, including risk appropriate perinatal care, injuries, physical activity, and smoking; however, the HIA does not apply a clear MCH lens to any of them. The recommendations have mostly general relevance for MCH populations and stakeholders, but similar to the assessment content, few MCH specifics are provided
Report Title: South Central Neighborhoods Transit Health Impact Assessment Database Title: Phoenix Light-Rail Transit Extension Year: 2015 Lead Organizations: Arizona Department of Health Services Decision-making Level: Local Target Sector: Transportation HIA Summary: This health impact assessment will inform the transit extension connecting South Phoenix, a disadvantaged neighborhood, to the existing light-rail line of the city Web link: http://www.pewtrusts.org/~/media/assets/external-sites/health-impact-project/maricopa-cnty-ph-2015-scnthia-report.pdf?la=en	Partial: Raising Special Kids, an organization working with children who have disabilities, was noted in the Acknowledgements section of the HIA report as a member of the Community Advisory Group. They are the only organization noted that has a specific MCH population focus; though several local health care and health services organizations were also noted, as was the local health department, who led the HIA through a pass-through grant from the state health department. Documentation does not clearly state if or how any of these stakeholders added a specific MCH perspective	Yes: The Executive Summary states: “While the health impacts on all residents were considered, the HIA focused on the unique needs of pregnant women” and “families with children or youth with special health care needs,” along with other population groups (p. 11). The HIA presents detailed data and analyses specific to these populations throughout	Though it does not explicitly address any specific NPMs, this HIA has content judged to be at least partially related to five (3, 7, 8, 11, & 15). Sample content is included below for 3, 11, & 15 NPM 3—Risk-Appropriate Perinatal Care (‘Partial’): This HIA reports that “the proposed transit corridor was found to have particular implications for…pregnancy and birth outcomes” (p. 12), going on to note that “births to study area residents are more likely to result in low-birth weight; premature deliveries; and higher rates of infant mortality,” adding that these “residents have a high rate of publicly funded births, but poorer birth outcomes” than others in the county (p. 33). Perhaps most relevant for this NPM, “births among residents of the study area are more likely to be to women with inadequate prenatal care;” however, no data are presented specific to perinatal care and percent of very low birth weight (VLBW) infants born in a hospital with a Level III + Neonatal Intensive Care Unit (NICU) (p. 33) NPM 11—Medical Home (‘Partial’): The HIA discusses regular access to health care through the lens of transit accessibility, or lack thereof, for residents of the study corridor (pp. 100–104). The report does not specify medical home explicitly, but this discussion of spatial access was judged to be a relevant precursor to the NPM. In terms of children or youth with special health care, this HIA sates: “Previous [Maricopa County Department of Public Health]-commissioned projects highlighted transportation challenges facing families with children/youth with special health care needs (CYSHCN), and MCDPH receives funding from the Arizona Department of Health Services to include CYSHCN in projects related to policy, systems, and environmental changes. The [HIA] project team classified [families with children/youth with special health care needs] as priority populations, those who would be most affected by the proposed transit project” (p. 47) NPM 15—Adequate Insurance (‘Partial’): The HIA mentions insurance coverage as part of pathways connecting transit expansion to health outcomes throughout the document, most specifically related to potential impacts of transit expansion on household transportation costs and on local economic development, both of which are demonstrated to influence residents’ access and use of coverage (pp. 46–48). However, no specific data are presented on coverage status for children	The HIA team “identified 41 specific recommendations for improving community health” (p. 12), and though most are targeted toward the transportation sector, some have direct bearing on MCH stakeholders and populations. For example, “[Arizona’s Health Care Cost Containment System (AHCCCS)] and Medicare should expand education and outreach to healthcare recipients on how to utilize their available benefits to offset medical-related transportation costs” (p. 78) and “expand categorical eligibility of Valley Metro Reduced Fare Program to include parents/caregivers of adults/youth/children with special health care needs; persons enrolled in AHCCCS, women enrolled in the WIC, and persons enrolled in the SNAP; and pregnant women” (p. 80)	High: Although only one specific MCH stakeholder is noted in the report, pregnant individuals and CYSHCNs are among the populations of focus throughout. This HIA has content relevant for five NPMs, including risk-appropriate perinatal care, injuries, physical activity, medical home, and adequate insurance. The HIA recommendations reflect this general focus on MCH and offer solutions relevant to MCH stakeholders in the region and the state
Report Title: Transitional Jobs Programs: A Health Impact Assessment Database Title: Transitional Jobs Program HIA Year: 2013 Lead Organizations: University of Wisconsin, Population Health Institute Decision-making Level: State Target Sector: Labor and Employment HIA Summary: An HIA to inform a decision on the status of the Wisconsin Transitional Jobs Program that shaped the 2013–15 biennial state budget. The HIA focused on the immediate health effects of changes in income related to employment, such as diet, alcohol and tobacco use, and family cohesion, as well as long-term outcomes including chronic disease, mental health, and child well-being Web link: http://www.pewtrusts.org/~/media/assets/2013/01/wisctransitionaljobsprograms_ahealthimpactassessment.pdf	Yes: The Wisconsin Department of Children and Families administers the transitional jobs program examined in this HIA and provided ongoing consultation throughout the project	Yes: The transitional jobs program examined in this HIA has a specific focus on families, and the HIA project logic model includes “family cohesion” and “child maltreatment” as key intermediate health indicators potentially impacted by the program and associated with health outcomes that include “improved child well-being” and “improved birth outcomes” (p. 2)	Though it does not explicitly address any specific NPMs, this HIA has content judged to be at least partially related to six (3, 6, 7, 8, 9, & 14). Sample content is included below for 3, 6, 9, & 14 NPM 3- Risk-Appropriate Perinatal Care (‘Partial’): Though the HIA does not include data on perinatal care, it does consider how the jobs program influences contextual factors for maternal risk. For example, “prenatal exposure to alcohol and tobacco results in negative birth outcome[s], including premature deliveries, sudden infant death syndrome, and decreased lung growth. Birth outcomes resulting from prenatal exposure to alcohol includes infants born with significantly lower birth weights, height and head circumference and brain damage such as fetal alcohol syndrome” (p. 40) NPM 6- Developmental Screening (‘Partial’): While not considering screening rates, this HIA does touch on topics relevant to child development. In exploring evidence from evaluations of other transitional jobs programs, the report notes that “researchers observed some lasting effects for the participants’ children including improvement in school progress, boys’ standardized test scores, positive expectations for future school performance, the quality of social relationships, and participation in extracurricular activities” (p. 20) NPM 9- Bullying (‘Partial’): The HIA does not address bullying directly, but it examines relationships between the transitional jobs program and behavioral problems in children. Among evidence supporting this connection, the assessment identifies "studies that have examined how social capital is reflected in children's health and educational outcomes,” which “suggest the effect is positive on behavior problems" (p. 43). The report also “notes that among victims of child maltreatment, psychological problems are prevalent and often manifest in aggressive behaviors towards both adults and peers" as part of a pathway connecting the jobs program to family cohesion (p. 44) NPM 14—Smoking (‘Partial’): Smoking is mentioned throughout the document, but the report does not give percentages on women smoking while pregnant or children who live in a home where people smoke. However, the HIA’s literature review found that “parental alcohol and tobacco use can negatively affect children’s health in a number of ways, including through postnatal exposure to alcohol and tobacco and through modeling which results in the increased likelihood of children using alcohol and tobacco" (p. 40)	Because the jobs program is administered by the state Department of Children and Families, all the recommendations could be viewed as relevant to MCH stakeholders and populations. One of the three high-level recommendations included in the Executive Summary is specific to MCH populations: to "assure priority in the [transitional jobs] program to applicants with children, while not making parenthood an eligibility requirement of the program" (p. 5). The list of detailed recommendations for mitigating potential negative health consequences of the program advises contractors implementing the program to "include training and supports on work and family balance and stress management" (p. 60)	High: This HIA clearly involved MCH stakeholders in the form of the state Department of Children and Families. The report also includes a detailed focus on how the jobs program potentially impacts mothers and children through a variety of pathways. The assessment presents content relevant to at least six NPM topics, including risk appropriate care, developmental screenings, injuries, physical activity, bullying, and smoking. The recommendations generated by this assessment are also clearly impactful for MCH populations and stakeholders, including the state Department of Children and Families, who administer the transitional jobs program

*’Report Title’ matches available source documentation. ‘Database Title’ and all other context/summary information from Health Impact Project Database
